# The effectiveness of self-regulated learning (SRL) interventions on L2 learning achievement, strategy employment and self-efficacy: A meta-analytic study

**DOI:** 10.3389/fpsyg.2022.1021101

**Published:** 2022-10-28

**Authors:** Jing Chen

**Affiliations:** Research Centre of Foreign Language Education, College of Foreign Languages, Huazhong Agricultural University, Wuhan, China

**Keywords:** self-regulated learning, meta-analysis, second/foreign language learning, self-efficacy, L2 learning achievement

## Abstract

Interventions that incorporated the teaching of self-regulated learning (SRL) strategies are assumed to be effective in improving students' second language (L2) performance as they support students' SRL activity and self-efficacy. Nevertheless, previous meta-analyses largely focused on students' language learning achievement, while neglecting the instructional effects on their SRL strategy use and self-efficacy, two key factors in SRL models. This meta-analytic study was thus conducted to address the gap by synthesizing the evidence of SRL interventions in influencing students' L2 learning achievement, strategy use, and self-efficacy. The largest effect was obtained for L2 learning achievement (*g* = 1.39), followed by self-efficacy (*g* = 0.45) and strategy use (*g* = 0.40). Moderator analysis revealed similar instructional effects on students of different age groups and education levels. The duration and intensity of intervention significantly moderated the effectiveness of SRL interventions in the L2 context, especially for strategy use and self-efficacy. The findings obtained in the current study could inform practitioners and researchers of the cumulative effects of SRL interventions in L2 classrooms and study design and student characteristics that moderate the instructional effectiveness.

## Introduction

Factors influencingacademic outcomes have always received great attention from researchers and practioners (Jansen et al., 2019). In the field of second/foreign language (L2/FL) teaching and learning, there is an increasing evidence suggesting the crucial role of self-regulated learning (SRL) in facilitating language learners' abilities to accomplish learning goals (e.g., Oxford, 2013; Teng and Zhang, 2020). SRL is described as a strategic, dynamic process whereby learners activate and sustain their cognitions, motivations, and behaviors toward the attainment of learning goals (Schunk and Zimmerman, 2007; Zimmerman and Schunk, 2011). Many researchers thus leveraged on SRL-strategies-based interventions with the aim to support learners' knowledge of SRL and their engagement in SRL activities, and positive effects on students' L2 learning achievements were reported (e.g., Graham et al., 2020b; Teng and Zhang, 2020; Bai and Wang, 2021; Teng, 2022). Furthermore, the experience of deploying SRL strategies to successfully complete tasks could convey to learners' beliefs of their competence in L2, thus positively affecting their self-efficacy (Schunk and Zimmerman, 1997; Bai and Guo, 2018). Empirical evidence indicating the positive relationships between the employment of SRL strategies and self-efficacy in L2 setting is abundant in the existing literature (e.g., Graham et al., 2020b; Sun and Wang, 2020; Teng, 2022). It is therefore assumed that SRL interventions are effective in fostering L2 learning achievement, SRL strategy use and levels of self-efficacy. Nevertheless, research that has synthesized the effects of SRL interventions on the three outcome variables is still lacking. The current meta-analytic study attempts to fill in the research gap and examines the intervention and participant characteristics as moderators to inform the design of future SRL interventions for L2 learning.

## Literature review

### Theoretical framework of self-regulated learning

SRL has been researched extensively during the past two decades and various models, definitions, and frameworks have been proposed (e.g., Winne and Hadwin, 1998; Pintrich et al., 2000; Zimmerman, 2000; Boekaerts and Corno, 2005). Despite the various theoretical bases, SRL generally includes the main components of setting goals, monitoring and managing learning process, orchestrating the use of strategies, and revising goals when needed (Zimmerman and Schunk, 2011; Andrade and Evans, 2012). Self-regulated learners are metacognitively, behaviorally, and motivationally active in their learning as they proceed through three cyclical stages of SRL including forethought, performance, and self-reflection (Zimmerman, 2000, 2013). They utilize a range of SRL strategies, such as cognitive, metacognitive, and motivation regulation strategies, as well as behavioral strategic knowledge during their engagement in learning (Kaplan et al., 2009).

A prerequisite to the active employment of SRL strategies is learners' self-efficacy, the beliefs in one's perceived competence to attain designated types of performance (Schunk and Ertmer, 2000; Zimmerman and Schunk, 2001). Self-regulatory skills are of limited value if one does not possess a strong sense of self-efficacy to engage and persist in the learning activity (Zimmerman, 2000; Schunk and Pajares, 2010; Schunk and Usher, 2012). Researchers thus posit that self-regulatory behavior and self-efficacy are integrated during learners' engagement in learning rather than distinct entities (Kaplan et al., 2009; Panadero, 2017; Graham et al., 2020b). For instance, Zimmerman's (2013) model of SRL describes a reciprocal relation between strategy use and self-efficacy, in which self-efficacy underlies the forethought phrase of goal setting and strategic planning and affect the efforts and attention devoted to the different phases of SRL learning (Zimmerman and Risemberg, 1997; Bruning et al., 2013; Bai and Wang, 2021). Specifically, in the forethought phrase, learners analyse the learning tasks, set up goals and plan their work; they also activate their motivational beliefs, including self-efficacy. In the following phrase, the performance phase, learners apply a range of cognitive strategies, monitor their learning process, and regulate their motivation to facilitate the completion of learning tasks. Lastly, in the self-reflection phase, students reflect on their learning process and make causal contributions between the strategies they used and their learning outcomes. Namely, they evaluate which strategies were effective and determine what they would do differently the next time. Moreover, learners who attributed learning outcomes to their efforts in strategy employment are more likely to possess a greater sense of control over their leaning process. A successful language learning outcome could help in the development of mastery experience, which could contribute to a sense of competency in undertaking similar tasks in the future, thus a higher level of language self-efficacy (Bandura, 1997; Graham et al., 2020b).

In all, models of self-regulation depict that self-efficacy and strategic behavior come together in the SRL process and there might be a reciprocal relationship between language self-efficacy, SRL strategy use and language learning achievement (Panadero, 2017; Graham et al., 2020b). The three variables are thus considered as the main outcome variables in this meta-analysis.

### Previous meta-analytic findings of SRL interventions

Meta-analysis, which summarizes the magnitude and directions of the effects obtained in a set of empirical research, is a useful approach to explore the effectiveness of instructional practices (Lipsey and Wilson, 2001; Graham et al., 2012). To quantify the variations in effects across studies and to obtain the overall effectiveness of SRL interventions, researchers have undertaken a number of meta-analyses and positive effects on students' academic performance, strategy use and motivation were found (e.g., Graham et al., 2012; Jansen et al., 2019; Sun et al., 2022). For instance, Jansen et al.'s (2019) study adopted meta-analytic structural equation modeling to investigate whether SRL activity mediated the effects of SRL interventions on achievement at the tertiary level. Their results revealed a positive effect of SRL interventions on university students' SRL activity (*d* = 0.50) and achievement (*d* = 0.49) across various academic subjects, which were classified as social sciences, formal sciences, applied sciences or a combination of them. A more recent study by Theobald (2021) provided further insights into the differential training effects of SRL interventions by synthesizing 49 studies conducted at the tertiary level in real classrooms. A three-level meta-analysis based on 251 effect sizes revealed positive, small to medium effects of extended SRL training programmes on students' academic performance (*g* = 0.37), motivational outcomes (*g* = 0.35), and the use of various strategies including metacognitive strategies (*g* = 0.40), resource management strategies (*g* = 0.39), and cognitive strategies (*g* = 0.32). The study also tested the instructional effects on specific aspects of motivation and reported a medium-sized effect for self-efficacy (*g* = 0.38).

The meta-analysis of SRL instruction for the specific domain of L2 learning, however, has received limited attention. Previous meta-analytic studies were conducted predominately in L1 contexts and many of them focused on writing instruction. Similar to findings in general learning contexts, positive findings were reported for language learning, specifically for learning to write. For example, Graham et al.'s (2012) study found that self-regulation strategy instruction significantly improved elementary grade students' written text quality (*d* = 1.17). Since the previous meta-analyses aimed to examine the effects of various writing instructions, they did not probe into self-regulation strategies-based instructions and hence did not examine factors that moderated the interventional effects. Recognizing the research gap, Sun and Wang (2020) performed a meta-analytic study of self-regulated strategies-based writing instruction to examine for possible significant moderators. A positive, large effect was reported for students' writing outcomes (*d* = 0.73); moderator analyses indicated that design features, such as the duration of intervention and whether the studies involved random assignment, did not have a significant effect on the instructional effects. Nevertheless, since Sun et al. (2022) meta-analytic study focused on the instructional effects on language learning achievement, behavioral (e.g., SRL strategy use) or affective outcomes (e.g., self-efficacy) were not examined.

Taken together, the limited number of reviews that synthesized the effects of SRL interventions on strategy use and self-efficacy, combined with the significance of the two variables in L2 contexts, leads to the current meta-analytic study. The current study also aims to examine potential moderators of SRL intervention effectiveness in L2 contexts. Two main groups of moderators, which are informed by the previous meta-analytic studies reviewed above, are examined in this study.

### Moderators of the effectiveness of SRL interventions

To provide information on the future design of effective SRL interventions and to identify learners who benefit the most from SRL interventions, two groups of moderators that concern intervention characteristics and participant characteristics are examined in this study.

The first group of moderators encompasses factors related to the design of SRL interventions. Firstly, theoretical frameworks that underpin the design of SRL interventions are coded as a moderator in this study. Previous meta-analytic studies showed that SRL interventions differed in their theoretical backgrounds and emphasized different aspects of SRL. Interventions were more effective if they were based on metacognitive theories than those that were underpinned by cognitive theories (Dignath and Büttner, 2008; Theobald, 2021). Therefore, this study examines theoretical frameworks as a moderator to investigate if the instruction of certain groups of strategies is more effective than others for L2 learning. Secondly, the moderating effects of the intensity and duration of intervention are analyzed in this study as mixed results were reported in previous meta-analytic studies. Some studies found that shorter interventions were more effective than longer ones (e.g., de Boer et al., 2014; Ardasheva et al., 2017), while others reported no significant effects of the duration on instructional effectiveness (e.g., Sun et al., 2022). As for the intensity of interventions, de Boer et al. (2014) operationalized it as the number of sessions per week and duration of each session. Meta-regression analyses showed that while the number of sessions per week produced no significant influence, the duration of each session had a significant, small influence on instructional effects. However, in an earlier work (Dignath and Büttner, 2008), instructional effects were found to increase with instructions with more sessions. Furthermore, many primary studies did not provide detailed descriptions of the schedules or duration of interventions and researchers therefore could not probe into this moderator (e.g., Theobald, 2021). This study thus attempts to examine the moderating effects of the intensity as well as the duration of intervention to add to previous findings. Quality of the study design is analyzed as the third moderator. Although previous studies showed that the quality of primary studies did not moderate instructional effects (e.g., Jansen et al., 2019; Sun et al., 2022), this moderator has not been examined for SRL interventions in L2 teaching. Knowledge about the moderating effects of methodological features could contribute to the improvement of future experimental design in L2 learning.

The second group of moderators involve student characteristics. Participants' mean age and their educational levels as examined to provide further information on which group of L2 learners particularly benefitted from SRL interventions. Previous studies that examined students' age and grade level obtained inconsistent findings. For instance, Vosniadou (2020) study found that university students struggled with learning as self-regulatory demands increased after the transition from high school to university. It is therefore postulated that students at the beginning of tertiary level would benefit from SRL interventions. However, other studies found no significant differences between students of different grade levels in terms of their benefits from SRL instruction (e.g., Theobald, 2021; Sun et al., 2022). Therefore, it is not readily clear whether students' educational level or age is related with their learning achievement.

## The current study

Given the inconsistencies in findings from the primary research and the research gaps identified in previous meta-analyses, the present study aims to use the meta-analytic methodology to explore the evidence accumulated on SRL interventions for L2 learning. Two research questions guided this study:

1. What is the effect of SRL interventions on students' L2 learning achievement, SRL strategy employment, and self-efficacy?
2. Do intervention characteristics and participant characteristics moderate the effects of SRL interventions?

### Literature search

To identify studies to be included in the meta-analyses, a systematic literature search was performed via electronic databases, including Scopus, ERIC, Web of Science, and PsycInfo. The search queries for SRL interventions in the contexts of second/foreign language learning included: (“SRL” OR “self-regulation” OR “self-regulated learning” OR “self-regulatory strategies” OR “metacognitive strategies” OR “metacognitive skills” OR “cognitive strategies” OR “learning strategies” OR “learning style” OR “time management” OR “resources management” OR “motivational strategies” OR “motivation”) AND (“second language” OR “foreign language” OR “language learning” OR “FL” OR “L2” OR “learning outcome” OR “achievement” OR “performance” OR “success”) AND (“intervention” OR “treatment” OR “training programme” OR “foster”). Following Jansen et al.'s (2019) procedures, the search terms were kept broad intentionally to prevent the omission of relevant studies. This research resulted in 1,392 hits, including peer-reviewed, published articles and unpublished thesis. As the literature that has never entered the publication process can increase the risk of bias (Chow and Ekholm, 2018), in the current study, only gray literature of unpublished studies (i.e., traceable using online databases) was included.

### Criterion for inclusion and exclusion

The titles and abstracts of the remained studies were further filtered based on a set of criteria for inclusion and exclusion, which was informed by previous meta-analytic studies on SRL interventions (e.g., Jansen et al., 2019; Theobald, 2021). Firstly, only interventional studies that targeted SRL and involved direct strategy instruction were included; studies that supported SRL indirectly, such as providing prompts without explicit instruction, were excluded. Also, only extended SRL interventions, which were conducted in classroom environments, were included; one-time instructional experiments were excluded. As the current study aimed to examine the overall effectiveness of SRL interventions, the restriction could enhance the comparability across the interventional studies (Theobald, 2021). Moreover, as the study targeted regular students, studies that involved gifted students or students with learning disabilities or behavioral disorders were excluded. Lastly, studies had to report at least one of the following outcome measures: students' second/foreign language performance (either overall language proficiency or specific language skills), SRL strategies (i.e., cognitive, metacognitive, resource management, or motivational regulation strategies), or self-efficacy levels.

**Figure 1:**
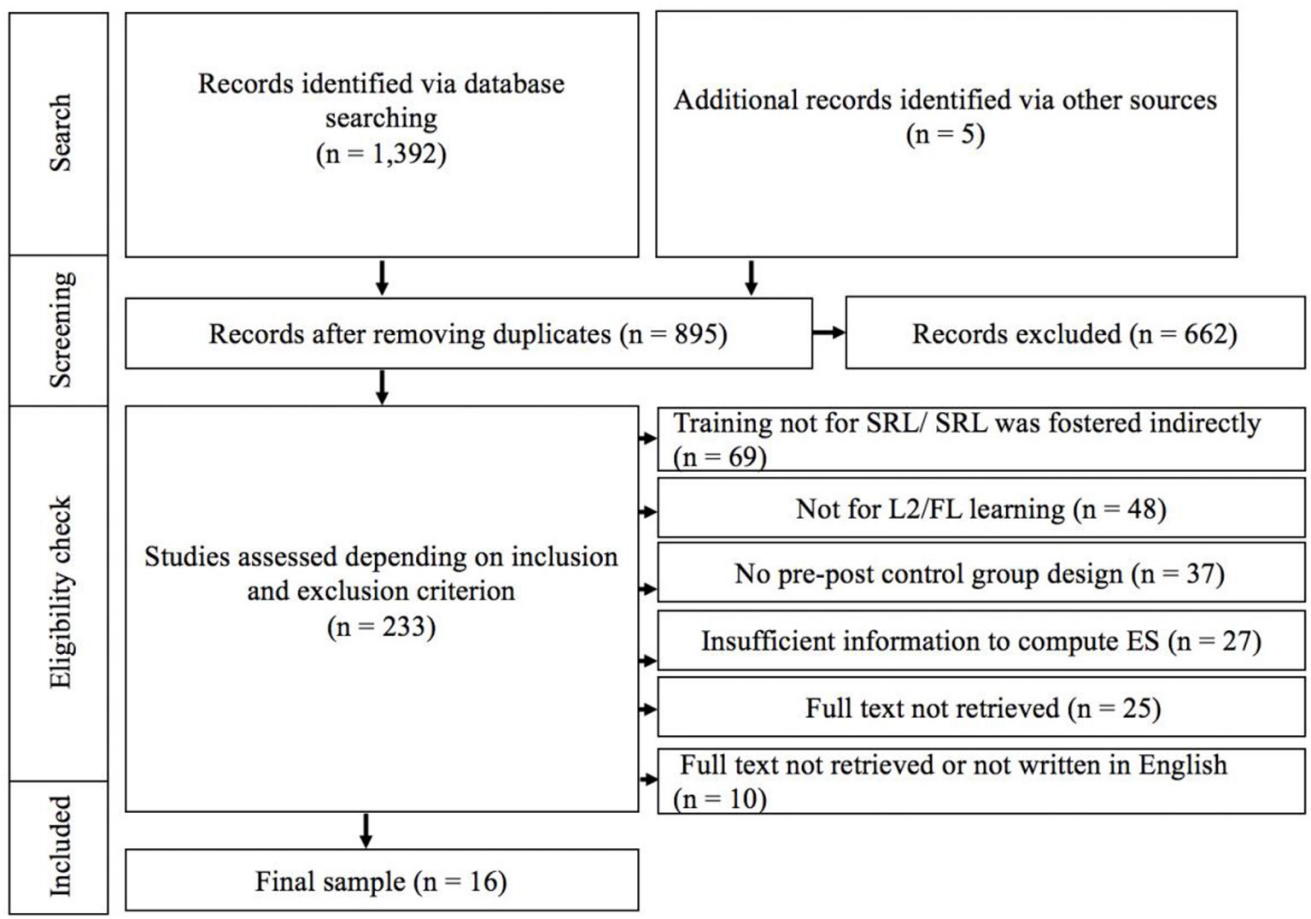
Overview of literature search process.

Figure 1 presents the flow diagram of the literature search process. A total of 233 studies was retained based on the criterion mentioned above. The common reasons for these articles to be removed included: (1) the studies targeted academic subjects rather than language learning; (2) the studies were conducted in a first language (L1) context; (3) the studies adopted an exploratory design and reported qualitative findings; (4) the studies did not include a control group. Only studies employing experimental or quasi-experimental pre-post control group designs were included to ensure the comparable methodological standard (Theobald, 2021). Studies that did not provide enough information to compute effect sizes were also excluded. In cases where information on means or standard deviations was absent, effect sizes could be computed by respective *F*-values of the interaction values of condition (experiment vs. control) and time (pretest vs. posttest). Studies whose sample sizes were too small per group (i.e., fewer than 10 participants) were also excluded. This was to ensure the normality of the effect sizes being included in the meta-analysis (Hedges and Olkin, 1985). The remaining studies, whose full texts were not available (e.g., conference abstracts) or the article was not written in the English language, were also excluded. The final set of studies included 16 articles.

### Coding of moderator variables

Each study was coded for intervention design and participant characteristics. Intervention design was first coded based on the theoretical framework to account for the differences in the focus of intervention content. Previous meta-analyses reported that theoretical backgrounds of SRL trainings moderated the effects on academic achievements as interventions could emphasize different aspects of SRL (e.g., Dignath and Büttner, 2008; Theobald, 2021). Informed by Theobald (2021), three types of interventional programmes were identified, including metacognitive, socio-cognitive, and cognitive interventions. An intervention was categorized as a metacognitive intervention if it focused on metacognitive strategies (e.g., planning, motoring, and evaluating) or metacognitive theories (e.g., Flavell, 1979) underpinned its instructional design (cf. Nguyen and Gu, 2013). An intervention was identified as a socio-cognitive intervention if it was informed by social-cognitive theories (e.g., Zimmerman, 2002) or targeted the use of resource management strategies combined with metacognitive reflection (cf. Macaro and Erler, 2008). An intervention was coded as a cognitive intervention if it focused on teaching cognitive strategies together with resource management strategies or was based on cognitive theories (cf. Abdolrezapour and Ghanbari, 2021).

The second moderator of intervention design concerns the duration and intensity of intervention. The duration of interventions was coded in weeks; the intensity of interventions was operationalized as the number of sessions per week and the duration of each session in hours (de Boer et al., 2014; Jansen et al., 2019). The third moderator of intervention characteristics is the quality of design. Following previous meta-analyses (Graham et al., 2012), the following indicators were coded: (1) random assignment of participants; (2) the establishment of treatment fidelity (e.g., classroom observation); (3) control of teacher effects (e.g., random assignment of teachers into conditions); (4) pre-test equivalence of the target outcome was evident in quasi-experimental designs; (5) little evidence of ceiling or floor effects for outcome variables in pretests for quasi-experimental designs; (6) little evidence of ceiling or floor effects for outcome variables in posttests (more than 1 standard deviation from the ceiling or floor); (7) total attrition was less than 10%; and (8) total attrition was less than 10% and equal attrition across conditions (less than 5%). Each indicator was scored for one (included) or zero point (not included) and a total score was calculated for each study. The maximum total scores are 6 and 8 points for experimental and quasi-experimental designs, respectively. Fourth, language skill was also included as a moderator of intervention design and was planned to be classified as writing, reading, other skills (listening, vocabulary learning, and L2 learning in general). Lastly, the time gap between the pre- and posttest was analyzed as a moderator and was coded in weeks. The way learning achievement was tested was categorized into standardized tests (e.g., standardized national test as in Graham et al. (2020b) study), or tests devised to align with research purposes (e.g., story reading and writing as in Teng, 2020b study).

Participant characteristics were first coded for educational grade level. Although the educational grade level of the included studies has three categories (i.e., tertiary, secondary, and primary), the number of studies at the secondary and primary level was limited. Therefore, the educational grade level of each study was coded as tertiary or other level. Participants' mean age (in years) was coded as the second moderator.

### Calculation of effect size

**Figure 2:**
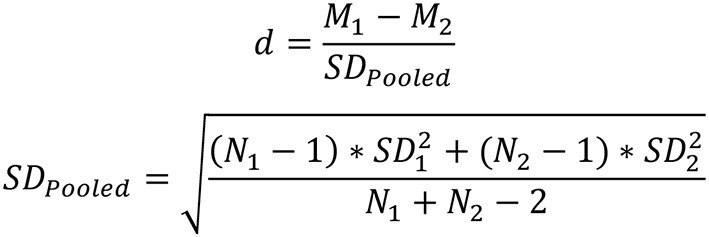
Formula for calculating Cohen's *d* for posttest-only control group design.

**Figure 3:**
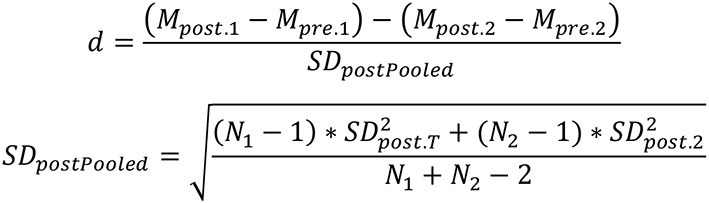
Formula for calculating Cohen's *d* for pretest-posttest control group design.

**Figure 4:**
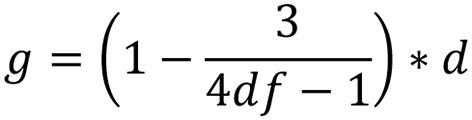
Formula for converting Cohen's *d* into Hedge's *g*.

**Figure 5:**
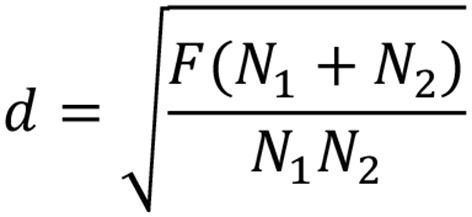
Formula for calculating *d* from *F*-test.

Informed by meta-analytic procedures in previous studies (e.g., Lipsey and Wilson, 2001; Boulton and Cobb, 2017; Sun et al., 2022), Cohen's *d* was computed through different procedures. For studies with a pretest-posttest-control-group design, Cohen's *d* was calculated by dividing the difference of mean change between intervention group and control group by the pooled pretest SD (Figure 2). Pooled pretest SDs were computed as the square-root of the two groups. For studies where only posttest data were reported and there were no pre-existing between-group differences, Cohen's *d* was calculated using post-standardized mean differences between the intervention and control groups (Figure 3). Cohen's *d* was then converted into Hedge's *g* to control for bias correction in the meta-analysis (Figure 4). The majority of the effect sizes was computed using the raw data, including mean, standard deviation, and sample size; in studies where the information was absent, Cohen's d was computed using *F*-values (Figure 5; Borenstein et al., 2009). Analyses were performed using R (R Core Team, 2019). Significance levels were set at.05 throughout the analyses.

To avoid inflating sample size and to meet the assumption of statistical independence, we used only one ES per study when calculating an average weighted ES for all studies included (Lipsey and Wilson, 2001). If an overall score for a variable (e.g., self-efficacy) was available, then the ES was calculated using this score. If only scores for sub-scales were available, an ES was computed for each subscale score and then averaged to produce a single ES. This is because including the same comparison on multiple outcomes violates the assumption of statistical independence and might render the standard errors and confidence intervals inaccurate (e.g., Turner and Bernard, 2006). Moreover, in studies where different intervention groups were compared with the same control group (e.g., Teng, 2019), comparisons were limited to the intervention group that closely aligned with the research purpose.

### Assessment of publication bias and outliers

Publication bias occurs when studies that are not included differ significantly from those that are included, instead of missing at random. For instance, studies with relatively large sample sizes and with significant results are more likely to be published than those with small samples and non-significant results, leading to bias in samples selected for inclusion in the current meta-analysis (Borenstein et al., 2009). Informed by previous meta-analyses, several statistical analyses, including the funnel plot with trim and fill, Orwin's fail safe N (Orwin, 1983), a Egger's correlation test examining the independence of variance and effect sizes (Egger et al., 1997), adding the sample size as a moderator and adding the article type (e.g., journal article or unpublished dissertation) as a moderator, were employed to identify potential presence of publication bias (Banks et al., 2012; Jansen et al., 2019; Graham et al., 2020a). The analyses were conducted for each of the three outcome categories using CMA v 3.3 (Borenstein et al., 2014). Furthermore, the effects sizes were examined for any potential outliers through the funnel plots and the impact of each study on the combined effect was assessed by calculating an effect size after removing it in CMA v 3.3.

## Results

### Descriptive results

The meta-analysis included a total of 24 effect sizes, originating from 16 studies which encompassed 1,780 participants. No outlier in the effect sizes was detected for each outcome variable. Approximately half of the effect sizes measured language performance outcomes (*n* = 13, 54.2%), 6 (25.0%) measured strategy use, and 5 (20.8%) measured self-efficacy. All of the studies were performed in an L2 context; the studies predominately targeted the learning of English as a foreign/second language (*n* = 21, 87.5%) and only three effects sizes computed from two studies involved the learning of other languages (i.e., French). As for the language skills, the majority of studies aimed to improve students' writing abilities (*n* = 10, 62.5%); the others focused on reading abilities (*n* = 3, 18.8%), listening abilities (*n* = 1, 3.1%), vocabulary learning (*n* = 1, 3.1%) and language learning in general (*n* = 1, 3.1%). A total of 10 (62.5%) studies was conducted with students at the tertiary level, and other studies in the primary (*n* = 5) or secondary (*n* =1) educational settings. Table 1 presents the coding information of the primary studies included in the meta-analysis.

**Table 1:** Coding information of studies included in the meta-analysis.

**References**	***n*** **participants**	**Outcome**	**Theoretical** **background **	***n*** **weeks**	***n*** **sessions/ week**	**Minutes/ session**	**Total amount (hours)**	**Quality score**	**Assignment**	**Time gap** **between pre-** **and posttest** **(weeks) **	**Type of test**	**Educational level**	**Age**	**Language skill**
Teng and Zhang (2020)	80	Writing performance; SRL strategies; self-efficacy	Socio-cognitive	20	1	90	30	6	Non-Random	20	Standard	Tertiary	18.8	Writing
Chen et al. (2022)	102	Writing performance	Socio-cognitive	8	1	90	12	5	Non-random	8	Devised	Tertiary	19.0	Writing
Chen et al. (2021)	56	Self-efficacy	Socio-cognitive	8	1	90	12	5	Non-random	8	Devised	Tertiary	19.0	Writing
Graham et al. (2020b)	529	Reading performance; SRL strategies; self-efficacy	Socio-cognitive	16	n/a	30	8	8	Random	28	Standard	Primary	11.5	Reading
Teng (2022)	59	Writing performance; self-efficacy	Socio-cognitive	16	1	90	24	4	Non-random	16	Devised	Tertiary	19.3	Writing
Lu et al. (2017)	120	Language performance; SRL strategies; self-efficacy	Socio-cognitive	6	1	120	12	6	Random	8	Standard	Tertiary	19.1	Learning in general
Abdolrezapour and Ghanbari (2021)	49	Listening performance; SRL strategies; self-efficacy	Cognitive	8	4	150	80	6	Random	8	Devised	Primary	15.5	Listening
Nguyen and Gu (2013)	91	Writing performance; SRL strategies	Metacognitive	8	n/a	60	9	4	Non-random	8	Devised	Tertiary	20.0	Writing
Teng (2019)	88	Writing performance;	Socio-cognitive	n/a	1.5	60	6	8	Random	n/a	Devised	Primary	11.5	Writing
Mizumoto and Takeuchi (2009)	146	Vocabulary test; SRL strategies	Metacognitive	10	1	90	15	6	Random	10	Devised	Tertiary	20.0	Vocabulary
Macaro and Erler (2008)	62	Reading performance; SRL strategies	Cognitive	56	1	20	9.3	4	Non-random	56	Devised	Secondary	12.0	Reading
Teng (2020a)	25	Reading performance	Metacognitive	n/a	n/a	60	10	6	Random	n/a	Devised	Primary	n/a	Reading
Teng (2016)	120	Writing performance; SRL strategies	Metacognitive	18	1	90	27	7	Random	18	Devised	Tertiary	n/a	Writing
Alshammari (2016).	51	Writing performance	Socio-cognitive	6	1	120	12	6	Random	6	Devised	Tertiary	21.5	Writing
Khodadady and Khodabakhshzade (2012)	58	Writing performance, SRL strategies	Metacognitive	16	n/a	n/a	Hours	6	Random	16	Standard	Tertiary	n/a	Writing
Teng (2020b)	144	Writing performance	Socio-cognitive	4	5	60	20	7	Non-random	4	Devised	Primary	n/a	Writing

n/a, information not available in the text.

### Publication bias

Three separate funnel plots were produced with the trim and fill method being applied to compare the obtained distribution of effect sizes and the predicted one (see Appendix 1). The Egger's regression test for funnel plot asymmetry was not significant for self-efficacy (*z* = 1.83, *p* = 0.16), strategy use (*z* = 2.44, *p* = 0.07), which suggest a symmetric distribution of effect sizes around the mean effect. However, the Egger's test was significant for L2 learning performance (*z* = 2.99, *p* = 0.01), suggesting funnel plot asymmetry. The trim and fill analysis was thus performed to locate missing studies that might be neglected due to publication bias (i.e., studies with non-significant results) and to re-impute an overall effect size for L2 learning achievement (Borenstein et al., 2011; Kang and Han, 2015). Under the random effects model, the effect size for L2 learning achievement was *g* = 1.32 (95% CI = [0.88, 1.76]). The trim-and-fill analysis showed that the re-imputed estimate was the same. Moreover, the results of Orwin's (1983) Fail-Safe N revealed that there would need to add 1,568 studies (L2 learning achievement), 205 studies (strategy employment), and 174 studies (self-efficacy) to bring the accumulative effect size to a trivial level of 0.01 for each of the outcome variable. The results suggest publication bias was not an issue for L2 learning achievement, as well as for the other two outcomes.

### Intervention effects by outcome measures

**Figure 6:**
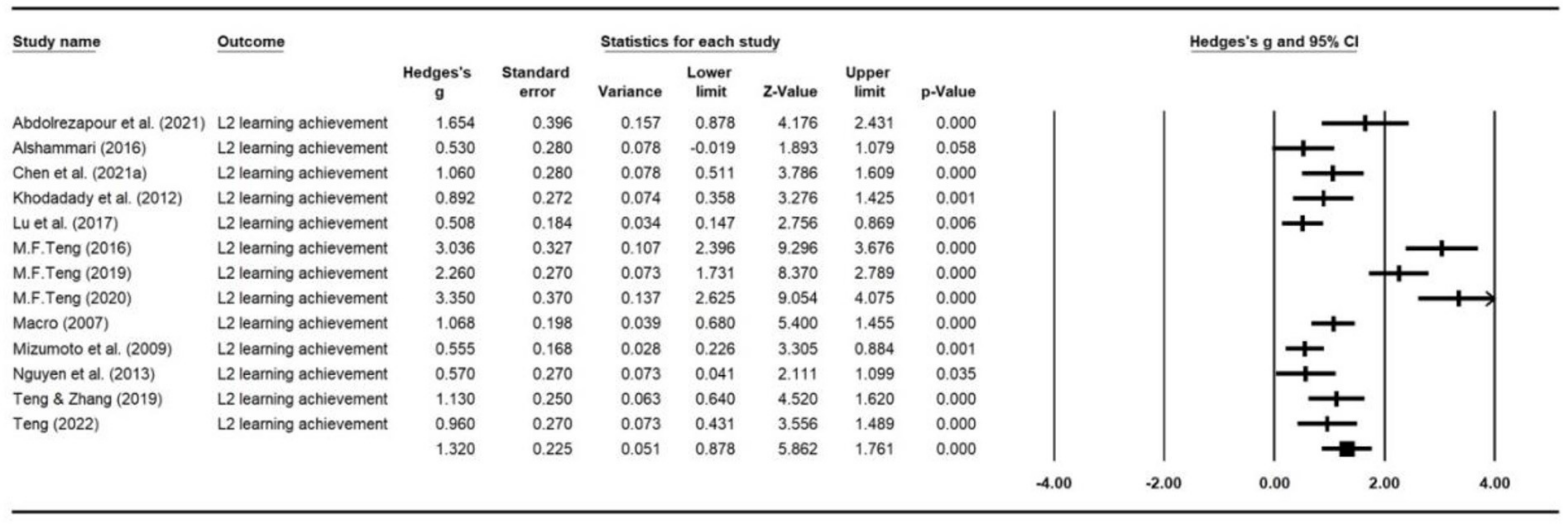
Forest plot of overall effect size estimates for L2 learning achievement.

**Figure 7:**
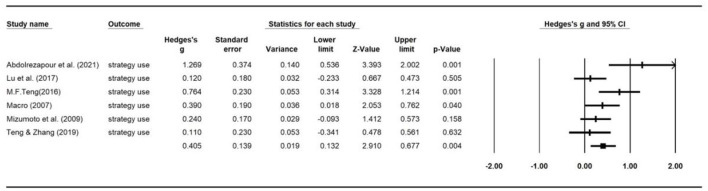
Forest plot of overall effect size estimates for strategy use.

**Figure 8:**
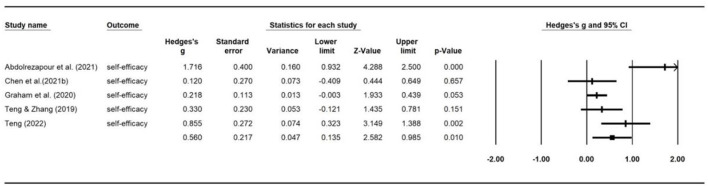
Forest plot of overall effect size estimates for self-efficacy.

The overall combined effect size based on 32 effect sizes was 0.72 ([0.62, 0.83], *z* = 13.16, *p* < 0.000). We used a weighted random effects model to aggregate ESs for language learning achievements, strategy use and self-efficacy. The forest plots of the overall effect size for each of the outcome variable are presented in Figures 6–8, respectively. As can be seen from the figures, the largest average effect size was obtained for language learning achievement (*g* = 1.32, *p* < 0.00), followed by self-efficacy (*g* = 0.56, *p* = 0.01), and strategy use (*g* = 0.41, *p* < 0.00). The results suggest that SRL interventions had a positive, large effect on L2 learning outcomes, and a medium sized effect on SRL strategy employment and levels of self-efficacy. A summary of the overall effect size for each outcome variable is presented in Table 2. The average effect sizes were then compared to examine whether they differed significantly between outcome categories. The average weighed effect size for L2 learning achievement was statistically larger than strategy employment (*Q*_between_ = 13.6, *p* = 0.001) and self-efficacy (*Q*_between_ = 27.2, *p* = 0.000), while the average effect sizes for strategy use and self-efficacy did not differ significantly.

**Table 2:** A summary of effect size estimation and heterogeneity statistics.

	**Effect size estimation**						**Heterogeneity**		
**Outcome**	***n***	***G***	**95% CI**	**SE**	***Z***	***p***	***Q***	***df***	***p***
L2 learning achievement	13	1.32	[0.88, 1.76]	0.23	5.86	0.00	122.24	13	0.00
Strategy use	6	0.41	[0.13,0.68]	0.14	2.91	0.00	12.47	5	0.03
Self-efficacy	5	0.56	[0.14;0.99]	0.22	2.58	0.01	17.20	4	0.00

### Results of moderator analysis

Substantial heterogeneity in effect sizes was detected for each of the outcome variable (see Table 2), indicating the necessity of moderator analysis to examine if effect sizes vary because of intervention characteristics and participant characteristics. Table 3 presents the results of moderator analysis for each outcome variable.

**Table 3:** Results of moderator analysis.

	**L2 learning achievement**			**Strategy use**			**Self-efficacy**		
**Moderator**	***k***	**Est. [CI]**	***p***	***k***	**Est. [CI]**	***p***	***k***	**Est. [CI]**	***p***
**Theoretical framework**									
a) Metacognitive	5	1.45 [53, 2.36]	0.00^*^	2	0.48 [−0.03,0.99]	0.07^+^	n/a		
b) Socio-cognitive	6	1.22 [0.55, 1.89]	0.00^*^	2	0.12 [−0.16,0.39]	0.41^+^	4	0.29 [0.12,0.47]	0.00^+^
c) Cognitive	2	1.26 [0.72, 1.80]	0.00^+^	2	0.77 [−0.08, 1.62]	0.08^+^	1	1.72[0.93, 2.50]	0.00^+^
**Duration of instruction**									
Weeks the instruction ran	12	−0.00 [−0.04,0.03]	0.90	6	−0.00 [−0.02,0.02]	0.93	5	−0.04 [−0.14,0.06]	0.43
Total amount of instruction in hours	11	0.01 [−0.02,0.03]	0.71	5	0.02 [0.00,0.03]	0.01^*^	5	0.02 [0.00,0.04]	0.01^*^
**Intensity of instruction**									
Frequency of session	11	0.42 [0.06,0.77]	0.02^*^	5	0.33 [0.04,0.62]	0.03^*^	5	0.46 [0.16,0.76]	0.00^*^
Intensity of each session	11	−0.74 [−1.93,0.44]	0.22	5	0.60 [−0.44, 1.64]	0.26	5	0.63 [0.02, 1.25]	0.04^*^
**Quality of design**	13	0.50 [0.07,0.93]	0.02^*^	6	0.08 [−0.25,0.41]	0.65	5	−0.10 [−0.45,0.26]	0.58
**Language skill**									
Writing	8	1.42 [0.76, 2.08]	0.00^*^	2	0.44 [−0.20, 1.08]	0.18^+^	3	0.43 [0.02,0.84]	0.04^+^
Other	5	1.17 [0.57, 1.77]	0.00^*^	4	0.39 [0.05,0.73]	0.02^+^	2	0.92 [−0.55, 2.38]	0.22^+^
**Assignment**									
a) Random	7	1.32 [0.64, 2.00]	0.00^*^	4	0.51 [0.09,0.93]	0.02^+^	2	0.92 [−0.55, 2.38]	0.22^+^
b) Non-random	5	1.39 [0.60, 2.17]	0.00^*^	1	0.11 [−0.34,0.56]	0.63^+^	3	0.43 [0.02,0.84]	0.04^+^
**Time gap between pre- and posttest**	12	0.00 [−0.04,0.03]	0.88	6	0.00 [−0.02,0.02]	0.90	5	−0.03[−0.09,0.03]	0.30
**Type of test**									
Standardized test	3	0.77 [0.51, 1.02]	0.00+		n/a			n/a	
Devised for the research	10	1.21[1.05, 1.37]	0.00^*^		n/a			n/a	
**Education level**									
a) Tertiary level	9	1.00 [0.58, 1.42]	0.00^*^	4	0.29 [0.02,0.57]	0.04^+^	3	0.43 [0.02,0.84]	0.04^+^
b) Non-tertiary level	4	2.06 [1.09, 3.03]	0.00^+^	2	0.77 [−0.08, 1.62]	0.08^+^	2	0.92 [−0.55, 2.38]	0.22^+^
**Age**	10	−0.10 [−0.23,0.02]	0.10	5	−0.05 [−0.14,0.05]	0.31	5	−0.19 [-1.5,0.54]	0.35

*k*, number of effect sizes; n/a, moderator variable not assessed for this variable.

df < 4; the results should not be interpreted.

* Significant at the level of .05.

#### Influence of intervention characteristics

Although all the primary studies encompassed the instruction of multiple SRL strategies, they differed in the relative focus of strategy instruction based on the theoretical framework. Following the operationalization scheme in previous meta-analysis (Theobald, 2021), SRL interventions were divided into three types: (1) metacognitive interventions; (2) socio-cognitive interventions; and (3) cognitive interventions. As presented in Table 3, the metacognitive interventions had the largest, significant effect size for L2 learning achievement (*g* = 1.45, *p* = 0.00), followed by socio-cognitive interventions (*g* = 1.26, *p* = 0.00) and cognitive interventions (*g* = 1.22, *p* = 0.00). However, the three types of interventions did not differ significantly in their effects on L2 learning achievements.

As the intensity of intervention was operationalized as the frequency of sessions per week and the intensity of each session in hours, meta-regression analysis was used with each of the two continuous variables as the predictor variable and effect size the dependent variable. As shown in Table 3, the frequency of sessions per week significantly moderated the effects on the three outcome variables (all *p* < 0.05), whereas the intensity of each session only moderated the effects on self-efficacy (*p* = 0.04). The results suggest that the instructional effects on all three outcome variables seem to increase with higher frequency of sessions per week, whereas the intensity of each session only positively influenced students' self-efficacy.

Similarly, the duration of instruction was also calculated as continuous variables which were analyzed using meta-regression. The results showed that the duration of intervention in weeks had no significant effects on either outcome variables (all *p* > 0.05), while the total amount of instruction time in hours had a significantly positive effect on strategy use and self-efficacy (both *p* < 0.05). The results indicated that while the weeks interventions ran did not moderate the instructional effects on any of the outcome variables, the total amount of instruction time in hours had a positive effect on both students' strategy use and self-efficacy.

As for the design of study, the results of meta-regression analysis showed that the quality of design significantly moderated the effects for L2 learning achievements (Z = 2.27, *p* = 0.02), with greater effect sizes for studies with higher scores for design quality. Both random (*g* = 1.40, *p* = 0.00) and non-random assignment (*g* = 1.32, *p* = 0.00) yielded statistically significant effect sizes. Nevertheless, the assignment method did not impact the effects sizes for L2 learning achievement (*Q* = 0.03, *df* = 1, *p* > 0.05).

The time gap between the pretest and posttest did not show a significant influence on the outcome variables (all *p* > 0.05). The way learning achievement was measured did not moderate the L2 learning achievement, either (*Q* = 1.37, *df* =1, *p* >0.05).

Lastly, the instructional effects did not vary between language learning skills. As there was only one study that targeted listening, vocabulary learning, and L2 learning in general, respectively, these skills were combined into one group. The results showed that SRL interventions that targeted writing skill did not differ from SRL interventions that targeted other language skills (*Q* = 0.30, *df* = 1, *p* > 0.05).

#### Influence of participant characteristics

Significant effect sizes were obtained for L2 learning achievement in both tertiary education and non-tertiary education. The educational level did not moderate the interventional effects on L2 learning achievements (*Q* = 3.59, *df* = 1, *p* = 0.06), suggesting that the effects estimated from studies at the tertiary level did not differ significantly from studies at other educational levels. Moreover, the analysis of meta-regression with participants' mean age as the independent variable returned a non-significant result for L2 learning achievement and strategy use, which are presented in Table 3. The results indicate that participants' age did not significantly moderate the instructional effects for any outcome variable. Nevertheless, the effects obtained for non-tertiary education should be interpreted with caution because of the limited number of primary studies (four studies).

## Discussion

The present meta-analysis tested the effectiveness of SRL interventions in L2 contexts. Results revealed a large effect for L2 learning achievement (*g* = 1.39), and small average effects for strategy use (*g* = 0.40) and self-efficacy (*g* = 0.45) (Plonsky and Oswald, 2014). The results suggest that SRL interventions effectively enhanced participants' L2 learning achievement, strategy employment, and levels of self-efficacy. Moderator analyses further revealed that the design of interventions and participant characteristics moderated the effectiveness of intervention. The results below are discussed separately for each outcome variable.

### Effectiveness of SRL interventions on L2 learning achievements

The positive results from this study add to previous meta-analytic findings (e.g., Jansen et al., 2019; Theobald, 2021; Sun et al., 2022) that indicated positive effects of SRL interventions on students' academic achievements. Specifically, similar to previous meta-analyses that revealed a large effect of SRL interventions for L1 learning (e.g., Graham et al., 2012), a large effect was also obtained for L2 learning achievements, which served as evidence in support of the effectiveness of SRL interventions for language learning in both L1 and L2 contexts.

Intervention characteristics were tested as moderators of the effect of SRL interventions on L2 learning achievement and none of them were found to be a significant moderator. Firstly, moderator analyses revealed that instructional effects did not differ significantly depending on the theoretical frameworks of the intervention. All three types of SRL interventions, including metacognitive, socio-cognitive and cognitive interventions, had a significant large effect on L2 learning outcome. The results are not in line with Theobald's (2021) meta-analytic study in which interventions based on metacognitive theories were more effective in improving students' academic performance than study skill trainings based on cognitive theories. A possible reason for the inconsistent finding is the difference in student population being included. Theobald's (2021) study focused on the tertiary level and university students exclusively, while the current study included participants across various levels including the primary, secondary, and tertiary level. For younger students, cognitive strategy instruction is crucial and especially effective as they have not yet internalized a repertoire of cognitive strategies they can implement during learning (Dignath and Büttner, 2008; Jansen et al., 2019). Nevertheless, for university students who have already acquired a strategy repertoire, metacognitive training on how, when and why to use a specific strategy might be more effective. In this study, all the metacognitive or socio-cognitive interventions were conducted at the tertiary level except one study (cf. Teng, 2019) while all the cognitive interventions were conducted with primary or secondary level students. Therefore, no significant differences between the three types of interventions were found as they probably targeted different age groups.

The study also found that the effect sizes estimated from studies without random assignment did not differ significantly from those with random assignment, indicating that the experimental design and quasi-experimental design had similar effects for L2 leaning achievements. The results are consistent with previous meta-analyses (e.g., Rakes et al., 2010; de Boer et al., 2014; Sun et al., 2022), where the potential between-group differences did not impact the effect size estimation when pretest-posttest differences were used. The results obtained in the current study, along with previous meta-analyses of SRL interventions (e.g., Sun et al., 2022), suggest that the quasi-experimental design can be an alternative where true experimental intervention is not feasible. It should be noted that the quality of design significantly impacted the effects on L2 learning achievement (*Z* = 2.26, *p* = 0.02). This finding suggests that designs with higher quality produced greater instructional effects on L2 learning outcome. Taken together, the findings from methods of assignment combined with the quality of design imply that non-random assignment could be applied only when other factors that might reduce the design quality were controlled.

The duration of interventions did not significantly influence the effects sizes for L2 learning achievement. Specifically, two indicators of the duration of intervention, weeks the intervention ran and total amount of instruction time in hours, did not moderate the effects on L2 learning achievement. This finding is in line with meta-analytic studies conducted in L1 contexts (e.g., Dignath and Büttner, 2008; Koster et al., 2015; Sun et al., 2022), which reported that the duration of interventions did not significantly impact participants' language learning outcomes. As for the intensity of interventions, the duration of each session in minutes did not produce a significant effect, either. Nevertheless, as most primary studies included in the study were conducted in real classes, the duration of each session predominately lasted between 1 and 2 h. The limited variance in the duration of each session could result in the insignificant result. The frequency of sessions per week however, significantly moderated the effects on L2 learning achievement. The results seem to suggest that interventions with more sessions per week were more effective than those with fewer sessions.

Furthermore, regarding the participant characteristics as moderators, the results showed that SRL interventions effectively enhanced participants' L2 learning achievements, irrespective of their educational level or mean age. Students' education level (i.e., tertiary or non-tertiary) did not significantly influence the instructional effects. The results resonate with Sun et al. (2022) study in which student's grade level was found to had non-significant effects on the effectiveness of SRL writing interventions. Even with primary-level students only, researchers (e.g., Dignath and Büttner, 2008; Graham et al., 2012) found no difference in the effects of SRL interventions between younger and older students. Nevertheless, it should be noted that in the current study, the educational level was broadly categorized as tertiary or non-tertiary level as the number for primary or secondary education was too limited to be analyzed as an independent category. The number of primary studies at non-tertiary levels was relatively small (i.e., *n* = 4), which might limit the generalization of this finding to a larger population.

### Effectiveness of SRL interventions on strategy use and self-efficacy in L2 contexts

A positive instructional effect was found for students' strategy use in this study, extending the extant literature on the effectiveness of SRL in facilitating strategy employment to the field of L2 learning. Similar to previous meta-analytic studies of SRL interventions in more general learning contexts (e.g., Jansen et al., 2019; Theobald, 2021), a medium sized effect was found for strategy use in the current study. Intervention characteristics and participant characteristics were also examined as moderators of SRL interventions on students' strategy use. Nevertheless, not the results of all combinations of moderator and outcome variables were interpreted because of the limited number of primary studies for certain combinations. From the moderators tested, the total amount of instruction time and frequency of sessions were found to significantly, positively moderate the effects for students' strategy use, indicating that SRL interventions with a greater intensity yielding greater effects on students' abilities to employ strategies in L2 learning. The findings support the hypothesis that instructional effects for strategies increase with the intensity of interventions (Jansen et al., 2019; Theobald, 2021). The primary studies in the meta-analysis were conducted predominately in real courses and the total amount of instruction time ranged from 9 to 80 h. Within this range, SRL interventions with more instruction time were more effective than those with less instruction time. The findings suggest that students need appropriate time to master higher-level self-regulation skills and interventions with low intensity may not be sufficient for students to develop such skills, especially when the intervention are conducted in real courses that should fall in the duration of one or two semesters. Nevertheless, readers should be cautious that the findings might not be generalized to SRL interventions conducted in lab settings and fall beyond the range of 9–80 h.

Similarly, SRL interventions also improved students' self-efficacy in L2 learning, which adds to the existing evidence of SRL in facilitating self-efficacy (e.g., Theobald, 2021). The total amount of instruction time and frequency of sessions positively moderated the instructional effects on students' levels of self-efficacy. This finding supported the postulation that despite the malleability of students' self-efficacy, it takes time for students to demonstrate a significant change in their level of self-efficacy (Manchón et al., 2007). Moreover, the duration of each session also had a significant effect on students' self-efficacy. This finding calls for interventions with longer sessions as well as greater intensity to sustain the effectiveness. It should be noted that moderators such as educational level and participants' mean age were tested in the current study, and the results were not interpreted because of the limited number of primary studies included in the analysis (fewer than four). It cannot be concluded in the current study if the intensity of instruction is the only attribute that moderated the SRL effects on students' strategy use and self-efficacy in L2 learning.

### Limitations and directions for future studies

The findings obtained in this study should be interpreted in light of several limitations. Firstly, publication bias might exist, especially in the examination of instructional effects on L2 learning achievement. As the number of studies that applied SRL theories in L2 teaching and learning is relatively limited, not many unpublished dissertations that met the inclusion criterion were included in this study, which might contribute to the existence of publication bias. Secondly, less confidence should be placed in the reliability of average effect sizes obtained from a small number of studies. For instance, only five SRL studies that examined self-efficacy were located and included for the analysis of effects on self-efficacy. The limited number of primary studies further prevented the identification of intervention or participant characteristics that could moderate the instructional effects on self-efficacy. Given that self-efficacy and strategic behavior might come together as described in models of self-regulation (i.e., Zimmerman, 2013), further studies should extend this line of research. Thirdly, the primary studies included in the meta-analysis predominately target the teaching of English language and only two studies involve the teaching of other languages (i.e., French). The effectiveness of SRL interventions on L2 learning achievement thus require further investigation which includes teaching languages other than English.

There are a number of gaps in research where more evidence is required. First, other participant characteristics such as their preexisting SRL skills and self-efficacy levels could moderate instructional effects and thus require further analysis. Previous research showed that L2 learners with high SRL skills and high self-efficacy did not benefit from the intervention as much as learners with low SRL skills and low self-efficacy (Graham et al., 2020b). It is possible that L2 learners who already possess the ability and willingness to effectively employ SRL strategies do not need much further support. Examining the characteristics that learners bring to classroom is necessary as it is a prerequisite to the design of individualized instruction (Graham et al., 2020b; Theobald, 2021). Moreover, measurement characteristics were not examined in the current study as there was too little diversity in this variable across studies. Nearly all primary studies employed a self-report questionnaire to measure strategy use and levels of self-efficacy. Previous meta-analyses (e.g., Jansen et al., 2019) reported that SRL interventions had significantly different effects on SRL activity depending on how SRL activity was measured. Stronger effects were obtained when it was measured with a counted measured (e.g., think-aloud in which students' verbalizations were coded) than being measured with self-report questionnaires. A possibility might be that when students were encouraged to verbalize their thoughts during learning, they were also prompted implicitly to adhere to SRL learning, thus the greater effects (Jansen et al., 2019). Studies in this line of research should utilize varied instruments such as log files or adopt a repeated measure of SRL activity to provide a more accurate measure of students' actual SRL behaviors.

## Conclusion and practical implications

In conclusion, this study provided preliminary evidence in support of the effectiveness of SRL interventions in enhancing students' L2 learning achievement, strategy use, and levels of self-efficacy, regardless of students' mean age and educational level. This finding leads to the practical implication that SRL interventions can be effective in supporting L2 learners' engagement in strategy use and achievement as well as in improving their self-efficacy for both university and younger students. Moreover, moderator analysis revealed that no intervention characteristics significantly affected instructional effects except for the intensity of intervention. Instructors are encouraged to implement SRL interventions with a greater intensity as they are more likely to produce greater effects on L2 learners' strategy use and self-efficacy. Many previous studies did not test the intervention intensity as a moderator as this information in many primary studies was not clearly reported to be coded (e.g., Jansen et al., 2019). This study added to previous findings on moderators in SRL instruction by showing the statistically significant role of intensity of instruction as indicated by the total amount of instruction time and frequency of sessions.

Since instructional effects did not differ significantly depending on theoretical backgrounds, L2 instructors have great freedom in their intervention design in terms of the theoretical framework (Jansen et al., 2019). For researchers in this line, quasi-experimental designs can be an alternative when true experiments are not feasible as no significant differences were found between the two methods. Nevertheless, the quality of intervention should be ensured in other aspects such as treatment fidelity or equivalence of pre-test performance, as the quality of design positively influenced the effects on L2 learning achievement. Researchers are also encouraged to consider individual differences in designing SRL interventions to identify L2 learners who particularly benefit from SRL interventions.

## Data availability statement

The original contributions presented in the study are included in the article/supplementary material, further inquiries can be directed to the corresponding author/s.

## Author contributions

JC completed the research design, data analysis, and manuscript drafting.

## Funding

This work was supported by a grant from the Fundamental Research Funds for the Central Universities for project 2662021WGQD001.

## Conflict of interest

The author declares that the research was conducted in the absence of any commercial or financial relationships that could be construed as a potential conflict of interest.

## Publisher's note

All claims expressed in this article are solely those of the authors and do not necessarily represent those of their affiliated organizations, or those of the publisher, the editors and the reviewers. Any product that may be evaluated in this article, or claim that may be made by its manufacturer, is not guaranteed or endorsed by the publisher.

## Figures and Tables

**Figure A1:**
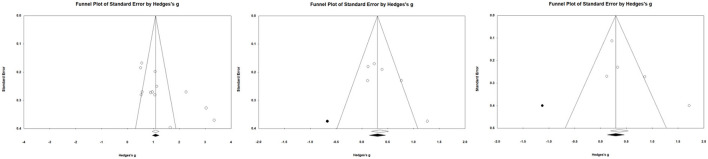
Funnel plot of standard error by Hedge's *g*.
